# Hereditary factors and exposures in childhood associated with adult chronic rhinosinusitis—the RHINE study

**DOI:** 10.1007/s00405-026-10358-x

**Published:** 2026-06-10

**Authors:** J. Bergqvist, L. Schiöler, C. Cramer, T. Gislason, E. H. Thorarinsdottir, M. Holm, R. Jogi, A. Johannessen, A. Malinovschi, L. Modig, C. Janson, V. Schlűnssen, J. Hellgren

**Affiliations:** 1 Department of Respiratory Medicine and Allergology, Sahlgrenska University Hospital, Gothenburg, Sweden; 2 Centre for Sleep and Wake Disorders, The Sahlgrenska Academy, University of Gothenburg, Gothenburg, Sweden; 3 Occupational and Environmental Medicine, School of Public Health and Community Medicine, Institute of Medicine, The Sahlgrenska Academy, University of Gothenburg, Gothenburg, Sweden; 4 Research Unit for Environment, Occupation and Health, Department of Public Health, Danish Ramazzini Centre, Aarhus University, Aarhus, Denmark; 5 Department of Sleep, Landspitali University Hospital, Reykjavik, Iceland; 6 Faculty of Medicine, University of Iceland, Reykjavik, Iceland; 7 Primary Health Care of the Capital Area, Reykjavik, Iceland; 8 Tartu University Hospital, Lung Clinic, Tartu University Hospital, Tartu, Finland; 9 Department of Global Public Health and Primary Care, University of Bergen, Bergen, Norway; 10 Department of Medical Sciences, Clinical Physiology, Uppsala University, Uppsala, Sweden; 11 Department of Epidemiology and Global Health, Umeå University, Umeå, Sweden; 12 Department of Otorhinolaryngology, Institute of Clinical Sciences, The Sahlgrenska Academy, University of Gothenburg, Gothenburg, Sweden; 13 Dept of Otorhinolaryngology, Alingsås Hospital, Region Västra Götaland, Alingsås, Sweden

**Keywords:** Chronic rhinosinusitis, Childhood, Asthma, Respiratory infections, Inner city living

## Abstract

**Background:**

The main aim of this study was to determine whether hereditary factors and exposures during childhood are associated with chronic rhinosinusitis (CRS) in adulthood.

**Methodology/principle:**

The Respiratory Health In Northern Europe (RHINE) study is a random population sample collected from seven centres in five countries during the period of 1990–1994. Data on CRS from the third follow-up in 2020 (RHINE 4) were examined in relation to previously reported hereditary factors and exposures during childhood.

**Results:**

In total, 7,686 subjects were included, with a mean age of 51.5 ± 7.1 years. The following items were associated with a significantly higher adjusted odds ratios (OR) for adult CRS: Serious respiratory infection at < 5 years of age (1.86, 95% CI 1.31–2.65); parental asthma (5.22, 95% CI 2.26–12.09); parental chronic obstructive pulmonary disease (COPD) (1.92, 95% CI 1.49–2.48); and living in the inner city compared with living on a farm as a child (1.50, 95% CI 1.05–2.15). Four or more members living in the household at 5 years of age (0.79, 95% CI 0.59–1.06), Childhood asthma (1.28, 95% CI 0.80–2.05), childhood furry pet (1.05, 95% CI 0.84–1.32), eating fish almost every day vs never (1.95, 95% CI 0.62–6.16), recurrent ear infections (1.14, 95% CI 0.89–1.47), and parental smoking (father smoker: 0.97, 95% CI 0.74–1.27; mother smoker; 0.90, 95% CI 0.62–1.32) were not significantly associated with adult CRS.

**Conclusions:**

Serious respiratory infection before the age of 5 years, parental asthma or COPD, and living in the inner city increases the risk for having CRS in adulthood. These factors should be taken into consideration when assessing airway vulnerability in children and their risk for chronic rhinosinusitis later in life.

**Supplementary Information:**

The online version contains supplementary material available at https://doi.org/10.1007/s00405-026-10358-x.

## Introduction

Chronic rhinosinusitis (CRS) is an inflammation of the sinonasal respiratory mucosa that causes persistent symptoms originating from the upper respiratory tract, resulting in a considerable negative impact on the health-related quality of life, reduced ability to work, and substantial health economic costs [11]. CRS is defined according to the European Position Paper on Rhinosinusitis and Nasal polyps (EPOS) as: nasal obstruction and/or secretion accompanied by facial pressure or pain, or loss of the sense of smell for more than 12 weeks [11]. The prevalence of questionnaire-identified CRS in adults living in Sweden is 9% [8] and 11% in Europe [13]. CRS is linked to other inflammatory airway diseases, such as allergic rhinitis, asthma and chronic obstructive pulmonary disease (COPD) [1, 10, 17]. It is also associated with gastroesophageal reflux disease (GERD), exposure to tobacco smoke, occupational sensitisers and irritants, as well as to damp conditions in the home [2, 6, 7, 25]. There are few studies on how hereditary and environmental exposures during childhood, when the respiratory immune system is maturing, are associated with CRS later in life. Chang et al. studied 772 subjects from 6 years of age to 22–32 years of age, and found that early onset sinusitis and recurrent upper respiratory tract infections in childhood were associated with an increased risk of suffering from sinusitis as an adult [5]. Perret et al. studied 3,364 subjects from age 6–7 years to a mean age of 53 years, in a prospective design using the EPOS definition of CRS,they found associations between childhood sinusitis, frequent childhood colds, episodes of tonsillitis, asthma in the mother, childhood asthma, and adult CRS [22]. As the results indicate that several environmental factors and exposures during childhood may be associated with adult-onset CRS, further study is warranted.

The present study is based on a large north European multi-centre cohort initiated in 1990, called The Respiratory Health in Northern Europe (RHINE) cohort. The main aim of this study was to investigate whether hereditary factors and exposures during childhood are associated with adult CRS (EPOS definition).

## Materials and methods

### Study population

**Fig. 1:**
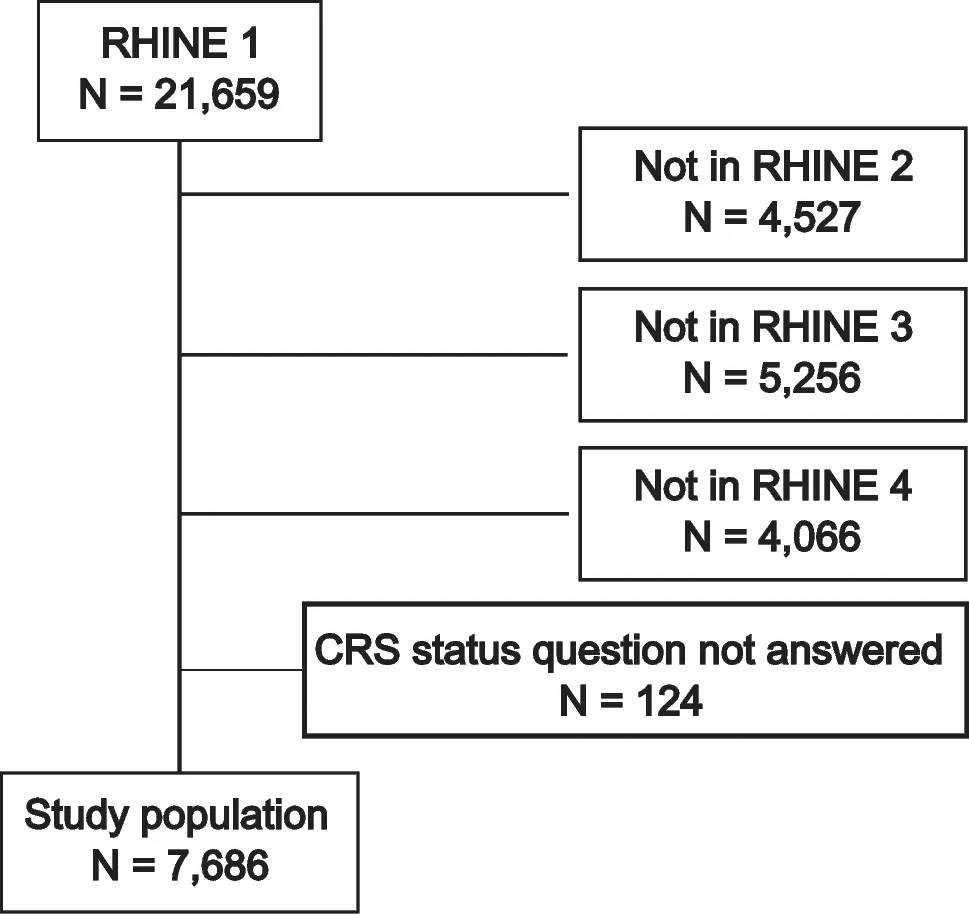
A flow diagram of the study population from the original RHINE study sample

The members of the study population were born in the period of 1945–1973 and participated in Stage 1 of the European Community Respiratory Health Survey (ECRHS I) in 1989–1992. They represent a random general population sample from seven centres in five countries (Aarhus, Denmark; Reykjavik, Iceland; Bergen, Norway; Tartu, Estonia; and Gothenburg, Umea, and Uppsala, Sweden) [21]. In the period of 1990–1994, the participants completed a postal questionnaire (RHINE 1), with follow-up questionnaires in 1999–2001 (RHINE 2), 2010–2012 (RHINE 3), and 2020–2023 (RHINE 4). The initial study population included 21,659 participants. The RHINE study aims to study airway diseases, such as CRS, asthma, COPD and associated risk factors. In the present study, we investigated whether retrospective self-reported exposure during childhood was associated with adult CRS reported in RHINE 4. A flow diagram showing the extraction of the study population from the original RHINE study sample is shown in Fig. 1. For this study, 7,686 participants were included.

### Definitions

CRS was defined as the presence of two or more CRS cardinal symptoms for ≥ 12 weeks, of which one must be either nasal blockage/obstruction/congestion or nasal discharge (anterior/posterior nasal drip), called “major symptom”, and/or additional symptoms, such as facial pain/pressure and/or a reduction in or the loss of smell, called “minor symptom”.

All subjects giving a positive answer to at least two major symptoms and/or one major and one minor symptom were defined as having CRS [11].

Items from the RHINE questionnaires related to hereditary factors and childhood exposures:

- *Living conditions as a child*: “What is the best description of the place where you lived most of the time under the age of 5 years? Options: 1) farm 2) village in the country; 3) small town; 4) suburb of city; or 5) inner city”.
- *Number of persons in the household at 5 years of age:* “How many persons lived in your home when you were 5 years old?”
- *Childhood furry pet*: “Was there any furry pets in your home when you were born?” and “Were there any furry pets in your home when you were a child?” Furry pets were: (one or more alternatives) dog, cat, other fur-bearing animals.
- *Childhood fish diet*: “How often did you eat fish as a child?” Options: Never, seldom, every week, several times a week, almost daily.
- *Serious respiratory infection at* < *5 years of age*: “Did you have a serious respiratory infection before the age of five years?”
- *Recurrent ear infections:* “Did you have recurrent ear infections as a child?”
- *Childhood asthma*: “Have you ever had asthma?” and “How old were you when you first suffered from asthma?” ≤ 18 years.
- Parental smoke:
  o *Mother smoker:* “Did your mother ever smoke on a regular basis when you were a child?”
  o *Father smoker:* “Did your father ever smoke on a regular basis when you were a child?”
- *Parental asthma, COPD*: Did your biological parents ever suffer from any of the following? father/mother: asthma or COPD

## Statistics

**Fig. 2:**
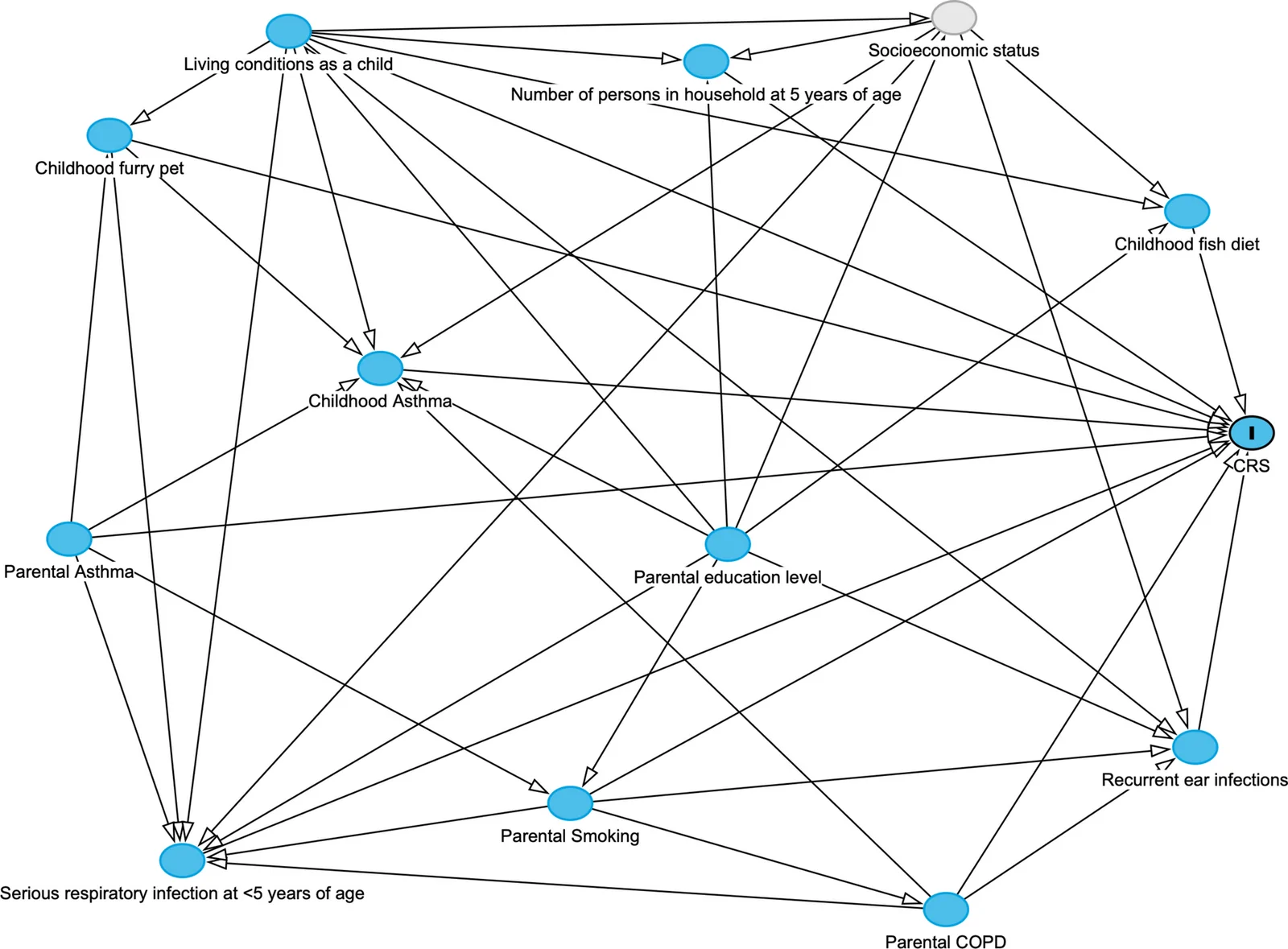
Directed acyclic graph (DAG) constructed based on potential associated factors. A minimum sufficient adjustment set for each model were chosen. Socioeconomic status indicates unobserved confounding

Descriptive statistics are presented as counts and percentages or as means and standard deviations for categorical or continuous variables, respectively. The prevalence rates of adult CRS (in RHINE 4) in relation to childhood hereditary factors and exposures were calculated and the corresponding p-values were calculated using Pearson’s chi-squared test. ORs for the prevalence of adult CRS were calculated using logistic regression. For each exposure, separate models were adjusted for a different set of a priori considered potential confounders (Table 1). In addition, all models were adjusted for centre, sex and age. The selected confounders were identified based on expert opinion and the limited available literature for this field. Potential associated factors were chosen by constructing a directed acyclic graph (DAG) in DAGitty (Textor, 2023) (Fig. 2) and selecting the minimum sufficient adjustment set for each model. Analyses were performed with the SAS version 9.4 statistical software (SAS Institute Inc., Cary, NC, USA).

**Table 1:** Inputs to the logistic regression models. In all the models, the outcome was adult chronic rhinosinusitis (CRS)

Model*	Model adjusted for the following exposures												
	Mother’s level of education	Father’s level of education	Number of persons in the household at 5 years of age	Childhood asthma	Childhood furry pet	Living conditions as a child	Childhood fish diet	Parental asthma	Parental COPD	Father smoker	Mother smoker	Serious respiratory infection at < 5 years of age	Recurrent ear infections
Living conditions as a child	X	X											
Serious respiratory infection at < 5 years of age			X	X	X	X	X	X	X	X	X		X
Childhood asthma			X		X	X		X	X	X	X	X	X
Father smoker	X	X						X			X		
Mother smoker	X	X						X		X			
Recurrent ear infections			X	X	X	X	X	X	X	X	X	X	
Parental asthma													
Parental COPD										X	X		
Childhood fish diet			X	X	X	X		X	X	X	X	X	X
Number of persons in the household at 5 years of age				X	X	X	X	X	X	X	X	X	X
Childhood furry pet						X		X		X	X		

^*^All models were adjusted for: centre, sex and age

In order to control for missing data, multiple imputation was performed (See supplement file Table S1, S2 and Figure S1) by using the fully conditional specification method [27] in SAS (PROC MI). Fifty imputed datasets were created, which we judged to be more than enough considering the amount of missing data. All variables that were used in any analysis were included. In addition, present smoking habits were included, as these may contribute information regarding the missing variables.

## Results

**Fig. 3:**
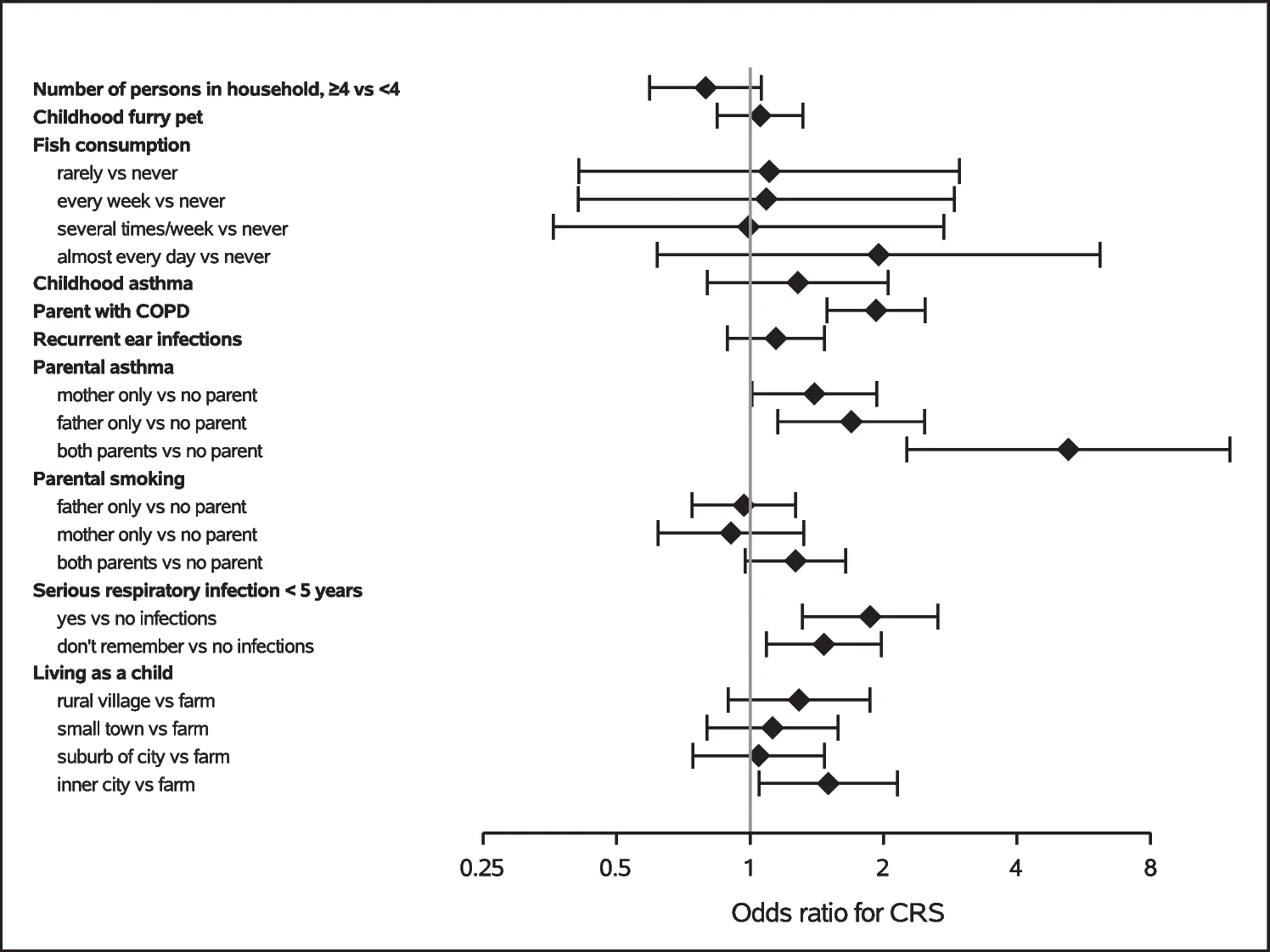
Odds ratio (OR) for the prevalence of adult chronic rhinosinusitis (CRS). Exposure specific models were used, in which each exposure was adjusted for a different set of a priori considered potential confounders (Table 1). In addition, all models were adjusted for centre, sex and age

The mean age in RHINE 4 was 51.5 ± 7.1 years and 53% were males. Table 2 shows the baseline data for the RHINE cohort, together with the CRS prevalence in Year 2020. The prevalence of adult CRS was significantly associated with *study site*, *eating fish as a child*, *Serious respiratory infection at* < *5 years of age* and *parental asthma and COPD*. The adjusted models of the ORs for having CRS in relation to hereditary factors and childhood exposures are shown in Fig. 3. *Parental COPD* (1.92, 95% CI 1.49–2.48), *parental asthma* (5.22, 95% CI 2.26–12.09), *living in the inner city* compared to *on a farm as a child* (1.50, 95% CI 1.05–2.15), and *serious respiratory infection at* < *5 years of age* (1.86, 95% CI 1.31–2.65) were all associated with a significantly increased OR for adult CRS. *Being four or more members in the family*, *Childhood asthma, childhood furry pet*, *eating fish almost every day vs never*, *recurrent ear infections* and *parental smoking* were not significantly associated with an increased OR for adult CRS. Multiple imputation did not alter the main results (Supplement file, Table S2, Figure S1).

**Table 2:** Baseline data for the RHINE cohort, together with chronic rhinosinusitis (CRS) prevalence in Year 2020. CRS was defined according to EPOS 2020

		Percent (*N*)	Prevalence of CRS (%)	*p*-value	*N* Missing
Age, years (mean ± SD)	51.5 ± 7.1				
Gender				0.24	-
	Men	46.8 (3,596)	5.3 (192)		
	Women	53.2 (4,090)	6.0 (244)		
Centre				0.0001	-
	Arhus	15.6 (1,197)	4.1 (49)		
	Reykjavik	13.6 (1,044)	6.9 (72)		
	Bergen	17.0 (1,310)	5.6 (73)		
	Gothenburg	12.5 (960)	5.4 (52)		
	Umea	15.1 (1,162)	6.3 (73)		
	Uppsala	17.4 (1,341)	4.3 (57)		
	Tartu	8.7 (672)	8.9 (60)		
Living conditions as a child				0.17	53
	Farm	15.9 (1,216)	5.1 (62)		
	Village rural	14.6 (1,115)	6.2 (69)		
	Small town	25.0 (1,909)	5.3 (102)		
	Suburb of city	29.2 (2,231)	5.3 (118)		
	Inner city	15.2 (1,162)	7.1 (82)		
Number of persons in the household at 5 years of age				0.14	157
	< 4	13.8 (1,038)	6.6 (69)		
	*≥* 4	86.2 (6,491)	5.5 (357)		
Childhood furry pet				0.78	150
	No	39.8 (3,000)	5.5 (166)		
	Yes	60.2 (4,536)	5.7 (258)		
Childhood fish diet				0.04	216
	Never	1.1 (83)	6.0 (5)		
	Rarely	24.7 (1,846)	5.6 (103)		
	Every week	53.8 (4,020)	5.3 (214)		
	Several times a week	18.2 (1,358)	5.7 (78)		
	Almost every day	2.2 (163)	11.0 (18)		
Serious respiratory infection at < 5 years of age				< 0.0001	204
	Do not remember	13.5 (1011)	8.0 (81)		
	No	79.5 (5,947)	4.8 (285)		
	Yes	7.0 (524)	10.3 (54)		
Recurrent ear infections				0.10	318
	No	79.4 (5,850)	5.3 (310)		
	Yes	20.6 (1,518)	6.4 (97)		
Childhood asthma				0.16	5
	No	95.6 (7,343)	5.6 (410)		
	Yes	4.4 (338)	7.4 (25)		
Parental smoking				0.39	378
	Mother	11.8 (865)	5.3 (46)		
	Father	26.9 (1,967)	5.2 (103)		
	Neither parent	34.7 (2,538)	5.5 (139)		
	Both parents	26.5 (1,938)	6.4 (124)		
Parental asthma				< 0.0001	22
	Mother	8.1 (618)	7.1 (44)		
	Father	4.7 (364)	8.5 (31)		
	Neither parent	86.8 (6,650)	5.3 (352)		
	Both parents	0.4 (32)	21.9 (7)		
Parental COPD				< 0.0001	22
	Neither parent	87.4 (6,700)	5.1 (345)		
	One Or Both	12.6 (964)	9.2 (89)

## Discussion

In this prospective, multi-centre, population-based study of almost 8,000 randomly selected subjects from five northern European countries, a history *of serious respiratory infection before the age of 5 years, living in the inner city as a child* and *parent asthma* or *COPD* were associated with an increased risk of CRS in adulthood. *Being four or more members in the family*, *Childhood asthma, childhood furry pet, eating fish during childhood, recurrent ear infections as a child* and *parental smoking* did not show higher ORs for adult CRS.

An important finding of the present study is that a previous *serious respiratory infection before the age of 5 years* is a risk factor for the development of CRS in adult life (Fig. 3). Serious respiratory infections in young children that results in hospitalisation include invasive infections that affect the entire airway, such as Respiratory Syncytial Virus (RSV) infection, which is known to be linked to the development of asthma. In addition, severe tonsillitis could also be interpreted as a serious respiratory infection if it leads to emergency visits or hospitalization. Previous studies have reported an association between childhood colds and upper respiratory tract infections and adult CRS [5, 22]. *Recurrent ear infections* were not related to an increased OR for adult CRS in the present study. This indicates that limited infections, such as ear infections, could have a weaker modulatory effect on the mucosal immune system than generalised infections of the upper and lower airways, such as the common cold or RSV infections.

It is possible for CRS and allergic rhinitis to co-exist, and both conditions show strong and independent associations with asthma. In the GA2LEN study, CRS without allergic rhinitis was associated with late-onset asthma, although a protective association with early-onset-asthma was detected. On the other hand, if the participant had both allergic rhinitis and CRS, the relative risks of developing both early-onset and late-onset asthma were dramatically increased [15]. In the present study, childhood asthma was not associated with CRS in adulthood. Similarly, Perret et al. [22] reported that *CRS diagnosed by a doctor* was associated with childhood asthma, whereas symptom-based CRS was not [22]. The lack of association with the broader symptom-based CRS definition is consistent with our findings and may reflect differences in disease severity and recall bias between the studies.

Early-life exposures that shape immune maturation may also contribute to the observed variability of risk. Early exposure to furry pets has been associated with both increased and decreased risks of allergic diseases, including asthma and allergic rhinitis [14, 19], In the present study, we found no such association between childhood exposure to furry pets and CRS in adulthood. This may reflect the fact that allergy can be involved in CRS but also in other immunological mechanisms.

An interesting finding here is that both *parental asthma* and *parental COPD* increase the risk for having CRS in adulthood. The presence of atopic disease, such as asthma, in parents is known to increase the risk of atopic disease in their children, especially when both parents share this genetic factor [28]. Therefore, it is not surprising that parental asthma increases the OR for adult CRS. The association observed for parental COPD is, however, unexpected. Both asthma and COPD are chronic inflammatory airway diseases, although their pathophysiologies differ. To the best of our knowledge, COPD in parents as a risk factor for adult CRS in offspring has not been shown previously. Awareness that a family history of asthma and COPD can affect the risk of developing CRS later in life may be relevant to early diagnosis of CRS and, thereby, contribute to effective prophylaxis and treatment.

Living in a rural environment as a child, especially on farms, has previously been shown to be protective against the development of allergic rhinitis [4, 23, 24]. In the logistic regression model, the OR for adult CRS was significantly higher in those subjects who reported *living in the inner city* as a child, as compared with those *living on a farm*. The present findings can be interpreted in relation to the hygiene hypothesis. This hypothesis, first formulated by Strachan in 1989 [26], proposed that declining family size and improved hygiene in westernised societies reduce childhood exposure to microbes, thereby increasing susceptibilities to allergic and chronic inflammatory diseases. In our study, *having four or more household members during childhood* was not associated with a lower OR for CRS in adulthood. *Living in the city* was also associated with a significantly increased OR for adult CRS compared to *living on a farm*. Farm environments are characterised by greater microbial diversity and bacterial endotoxin exposure, which have previously been shown to protect against allergic rhinitis and asthma [9]. Similarly, larger households likely entail more-frequent microbial exposures through siblings, thereby modulating immune maturation. Consistent with this, a recent review identified being an only child as a risk factor for allergic rhinitis and, potentially, also for CRS [18].

Growing up in inner-city environments may involve lower levels of exposure to microbes, combined with higher levels of exposure to pollutants and endotoxins in the inhaled air [12]. Moreover, children living in the in the inner city may have fewer opportunities for outdoor activities in a less-polluted environment than those living in a farm environment. Interestingly, *Serious respiratory infection before the age of 5 years* increases the risk of adult CRS. While this appears to run contrary to the hygiene hypothesis, it may represent a different mechanism, whereby a severe infection early in life induces immune dysregulation or structural airway changes rather than protective immune priming. Thus, while our results partly corroborate the hygiene hypothesis, they also highlight that the types and severity levels of childhood exposures are crucial in determining long-term outcomes.

Regular intake of fish has been associated with lower risk of allergic disease [20]. In this study, *regular intake of fish* was surprisingly associated with an increased prevalence of adult CRS. However, in the adjusted model, it did not affect the OR of having CRS in adulthood. Exposure to tobacco smoke has been shown to be a risk factor for CRS in adults [11]. Therefore, it is possible that tobacco smoke exposure in the domestic milieu during childhood is also a risk factor. However, this study showed no such increased risk.

### Limitations

A strength of the present investigation is that RHINE is a multi-centre study involving seven different centres in five countries, with a cumulative population of 23 million (2024). While the study conducted by Perret and colleagues was a single-centre study with an underlying population of around 600,000 [22], their results are in agreement with those of the present study.

The response rate in this study was 35% (7,686 participants of the original 21,659) and well in accordance with other well-known long-term respiratory health studies such as ECRHS [16]. In the previous follow-up (RHINE 3), we showed that baseline prevalence of several respiratory symptoms was higher in participants lost to follow-up compared to long-term participants, although the exposure-outcome associations remained unchanged. This indicates that the results remain valid despite the loss of participants to follow-up [16]. However, it also indicated that the prevalence of respiratory conditions, including, CRS,asthma and COPD may be underestimated in the present study. Another population-based cohort from Sweden reported that participation rates are often higher among participants with higher socioeconomic status [3]. Although such data are not available in the RHINE cohort, a lower response rate may contribute to further underestimation of both parental smoking and respiratory conditions such as CRS, COPD, and asthma, as all these variables are associated with lower socioeconomic status. In the RHINE study, the participants provided retrospective self-reports as adults on exposures and hereditary factors during childhood. This introduces a risk of recall bias for both exposures and outcomes. In addition, the question regarding serious respiratory infection is relatively broad, which may allow for different interpretations and thereby introduce reporting-bias. In the studies conducted by Chang et al. [5] and Perret et al. [22], the childhood exposures were based on answers that the parents gave at a singular time-point, when their child was between 6 and 7 years of age. The risk of “recall bias” must be considered, and based on the fact that the answers given by the parents on a single occasion concerning overall childhood exposures and hereditary factors, accuracy may be sub-optimal. It may be that it is better that interpretations are made over a longer observation period by the subjects themselves in adult life, as in the RHINE study.

EPOS has defined CRS according to the symptoms recorded in epidemiological studies of large cohorts conducted using questionnaires, which were used here. However, in the absence of a clinical nasal examination, it cannot be excluded that CRS has been over-diagnosed in this study. Asthma is also diagnosed in the clinic based on symptoms and a clinical examination and supported by spirometry. However, neither clinical examinations nor spirometry tests were included in this study. In addition, self-reported asthma and COPD can be overlapping in the absence of a clinical assessment.

## Conclusion

In this study of a large northern European cohort, a history of *serious respiratory infection before the age of 5 years, living in the inner city as a child, and parental asthma or COPD* were all associated with CRS in adulthood. Children at risk and their parents should be informed of the increased risk of developing CRS in adulthood, and further studies of preventative measures are warranted.

## Supplementary Information

Below is the link to the electronic supplementary material.

Figure S1 (PDF 63 KB)

Table S1 (DOCX 18 KB)

Table S2 (DOCX 21 KB)

## Data Availability

The data that support the findings of this study are not openly available due to reasons of sensitivity and are available from the corresponding author upon reasonable request.
