# Supplementary material for: Hereditary factors and exposures in childhood associated with adult chronic rhinosinusitis—the RHINE study
Source: Eur Arch Otorhinolaryngol. 2026 Jun 10;283(9):5757–65. doi: 10.1007/s00405-026-10358-x (PMC13615107; doi:10.1007/s00405-026-10358-x)
Supplement: Supplementary file 1 [file 405_2026_10358_MOESM1_ESM.pdf]

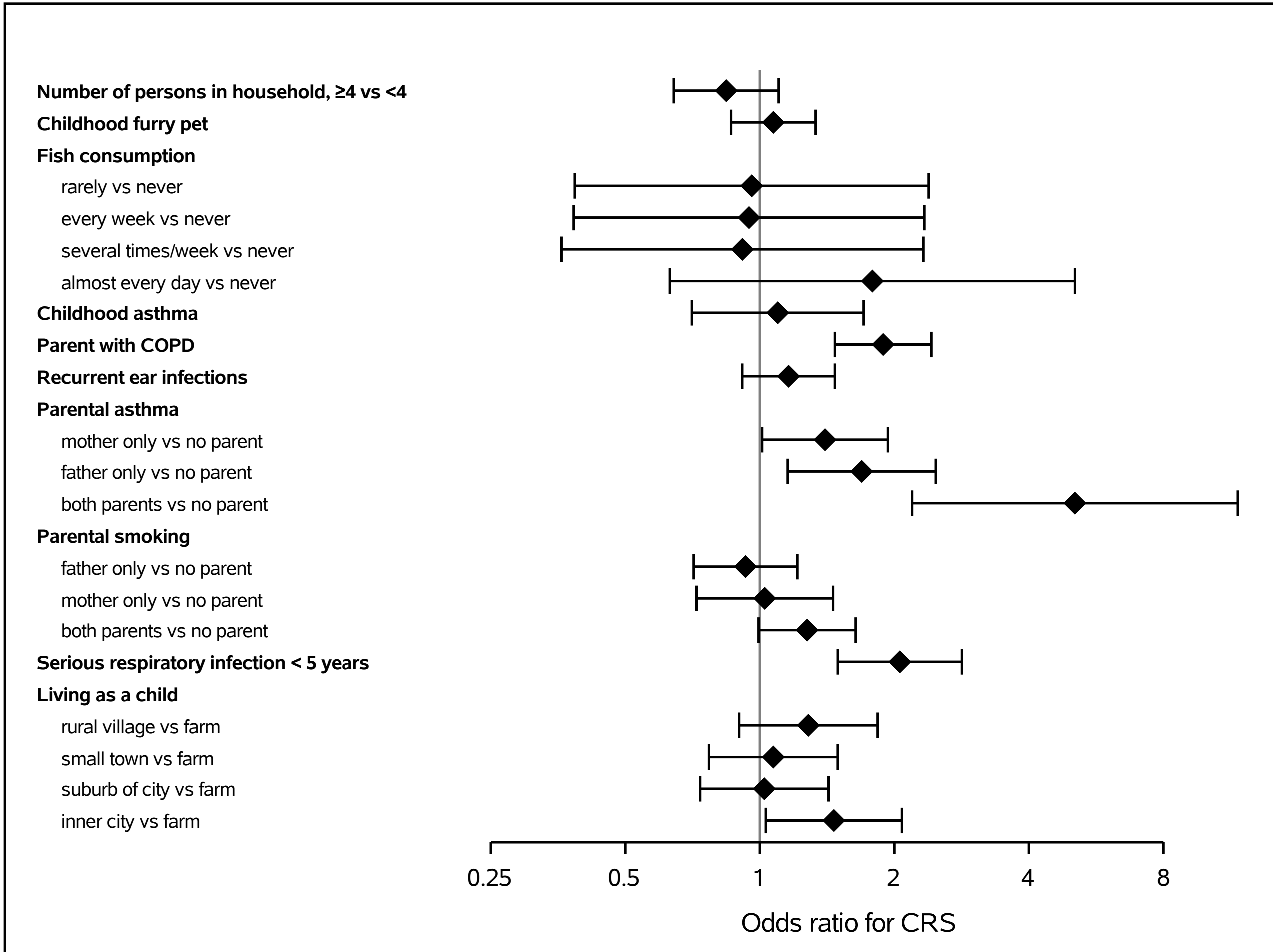

Figure S1. Odds ratio (OR) using multiple imputation for the prevalence of adult chronic rhinosinusitis (CRS). Exposure specific models were used, in which each exposure was adjusted for a different set of a priori considered potential confounders (Table 1). In addition, all models were adjusted for centre, sex and age
