# Supplementary material for: Hereditary factors and exposures in childhood associated with adult chronic rhinosinusitis—the RHINE study
Source: Eur Arch Otorhinolaryngol. 2026 Jun 10;283(9):5757–65. doi: 10.1007/s00405-026-10358-x (PMC13615107; doi:10.1007/s00405-026-10358-x)
Supplement: Supplementary file 2 [file 405_2026_10358_MOESM2_ESM.docx]

| **Item** | **N** |
| --- | --- |
| *Living conditions as a child* | 7312 |
| *Serious respiratory infection at <5 years of age* | 6704 |
| *Childhood asthma* | 6704 |
| *Parental smoke* | 7014 |
| *Parental asthma* | 7664 |
| *Recurrent ear infections* | 6704 |
| *Parental COPD* | 7295 |
| *Childhood fish diet* | 6704 |
| *Childhood furry pet* | 7129 |
| *Number of persons in the household at 5 years of age* | 6704 |

Table S1. Number of observations used when controlling for missing data using multiple imputation for all the exposures in the study
