# Supplementary material for: Hereditary factors and exposures in childhood associated with adult chronic rhinosinusitis—the RHINE study
Source: Eur Arch Otorhinolaryngol. 2026 Jun 10;283(9):5757–65. doi: 10.1007/s00405-026-10358-x (PMC13615107; doi:10.1007/s00405-026-10358-x)
Supplement: Supplementary file 3 [file 405_2026_10358_MOESM3_ESM.docx]

Table S2. Odds ratio (OR) using multiple imputation for the prevalence of adult chronic rhinosinusitis (CRS). Exposure specific models were used, in which each exposure was adjusted for a different set of *a priori* considered potential confounders. In addition, all models were adjusted for centre, sex and age.

| **Item** | **Model** | **Odds ratio (OR)** | **Lower CI** | **Upper CI** | **P-value** |
| --- | --- | --- | --- | --- | --- |
| *Living conditions as a child* | inner city vs farm | 1.46459 | 1.03135 | 2.0798 | 0.0330 |
|  | suburb of city vs farm | 1.02338 | 0.73503 | 1.4248 | 0.8912 |
|  | small town vs farm | 1.07215 | 0.76945 | 1.4939 | 0.6806 |
|  | rural village vs farm | 1.28395 | 0.89854 | 1.8347 | 0.1699 |
| *Serious respiratory infection at <5 years of age* | Serious respiratory infection < 5 years | 2.05762 | 1.49444 | 2.8330 | <.0001 |
| *Parental smoke* | both parents vs no parent | 1.27590 | 0.99358 | 1.6384 | 0.0562 |
|  | mother only vs no parent | 1.02577 | 0.72161 | 1.4581 | 0.8872 |
|  | father only vs no parent | 0.92878 | 0.71096 | 1.2133 | 0.5879 |
| *Parental asthma* | both parents vs no parent | 5.07141 | 2.19127 | 11.7371 | 0.0001 |
|  | father only vs no parent | 1.69051 | 1.15417 | 2.4761 | 0.0070 |
|  | mother only vs no parent | 1.39920 | 1.01178 | 1.9350 | 0.0423 |
| *Recurrent ear infections* | Recurrent ear infections | 1.15946 | 0.91314 | 1.4722 | 0.2246 |
| *Parental COPD* | Parent with COPD | 1.88778 | 1.47244 | 2.4203 | <.0001 |
| *Childhood asthma* | Childhood asthma | 1.09667 | 0.70455 | 1.7070 | 0.6827 |
| *Childhood fish diet* | almost every day vs never | 1.78620 | 0.62895 | 5.0727 | 0.2760 |
|  | several times/week vs never | 0.91418 | 0.35981 | 2.3227 | 0.8504 |
|  | every week vs never | 0.94565 | 0.38312 | 2.3342 | 0.9035 |
|  | rarely vs never | 0.95900 | 0.38533 | 2.3867 | 0.9283 |
| *Childhood furry pet* | Childhood furry pet | 1.07193 | 0.86209 | 1.3328 | 0.5320 |
| *Number of persons in the household at 5 years of age* | Number of persons in household, ≥4 vs <4 | 0.84088 | 0.64183 | 1.1017 | 0.2086 |
